# Supplementary material for: The CRISPR/Cas9 Minipig—A Transgenic Minipig to Produce Specific Mutations in Designated Tissues
Source: Cancers (Basel). 2021 Jun 16;13(12):3024. doi: 10.3390/cancers13123024 (PMC8234985; doi:10.3390/cancers13123024)
Supplement: Supplementary file 1 [file cancers-13-03024-s001.zip › cancers-1240717-supplementary.pdf]

cancers

MDPI

Supplementary Materials: The CRISPR/Cas9 Minipig—A Transgenic Minipig to Produce Specific Mutations in Designated Tissues

Martin Fogtmann Berthelsen, Maria Riedel, Huiqiang Cai, Søren H. Skaarup, Aage K.O. Alstrup, Frederik Dagnæs-Hansen, Yonglun Luo, Uffe B. Jensen, Henrik Hager, Ying Liu, Henrik Callesen, Mikkel H. Vendelbo, Jannik E. Jakobsen and Martin Kristian Thomsen

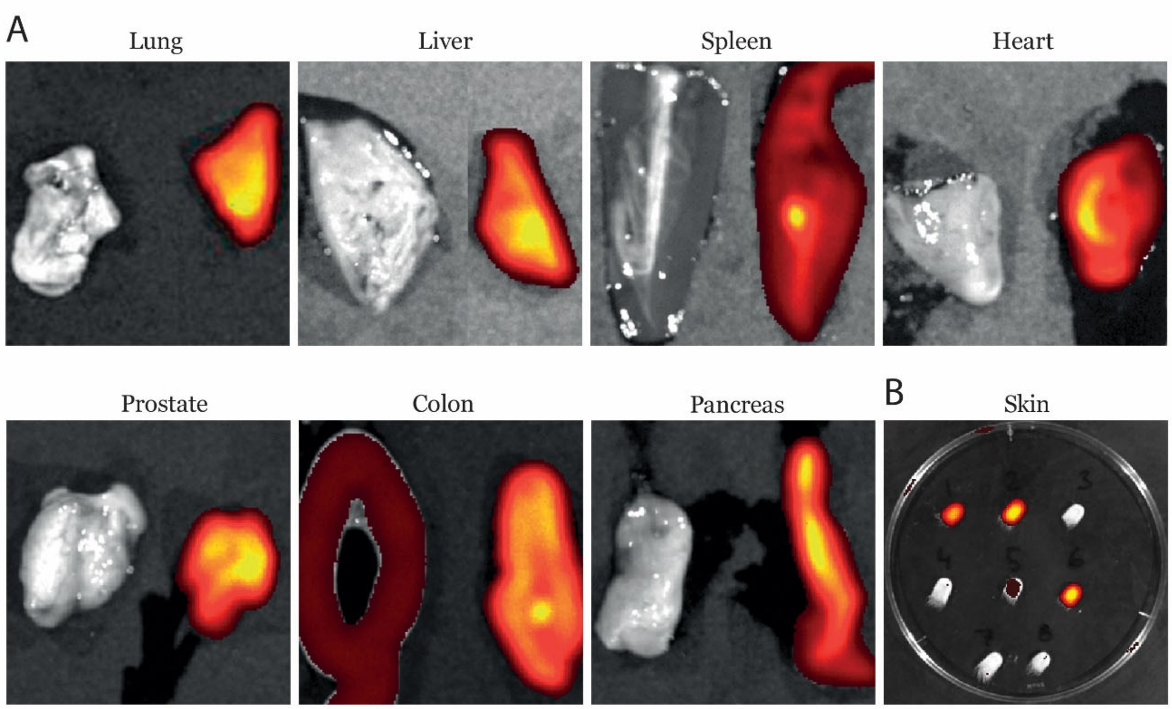

Figure S1. Transgenic expression. (**A**) IVIS scanning of tissues biopsies from a euthanized Cas9 pig verified transgene RFP expression in major organs. As a negative control tissue biopsies from a Danish Landrace pig were used. (**B**) IVIS scan of ear biopsies from a F1 litter.

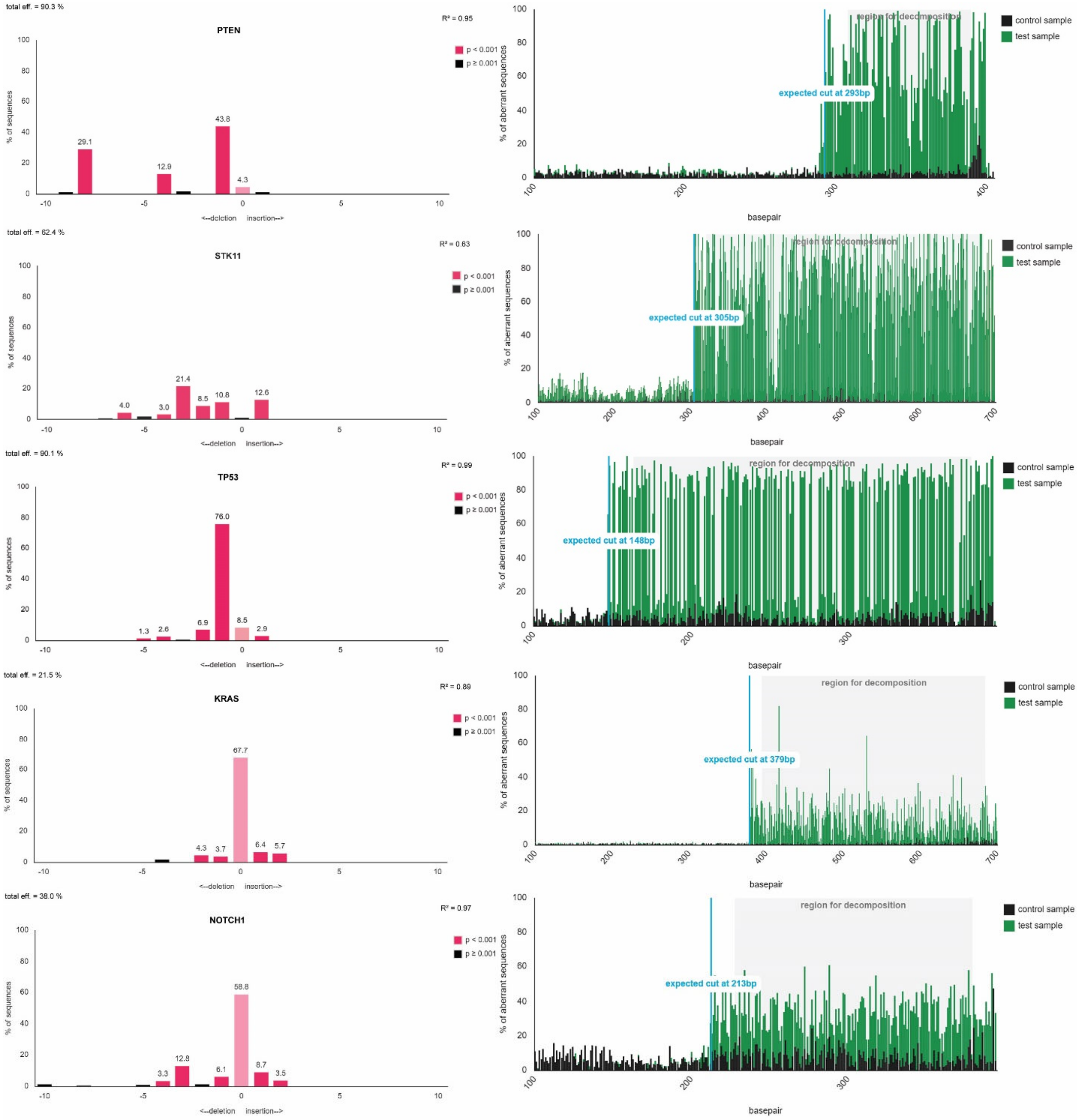

Figure S2. Validation of sgRNA in porcine fibroblast. Porcine fibroblasts transfected with the specified sgRNAs to determine guide efficiency. Mutations efficiency was assessed by TIDE analysis: Tracking of Indels by Decomposition software.

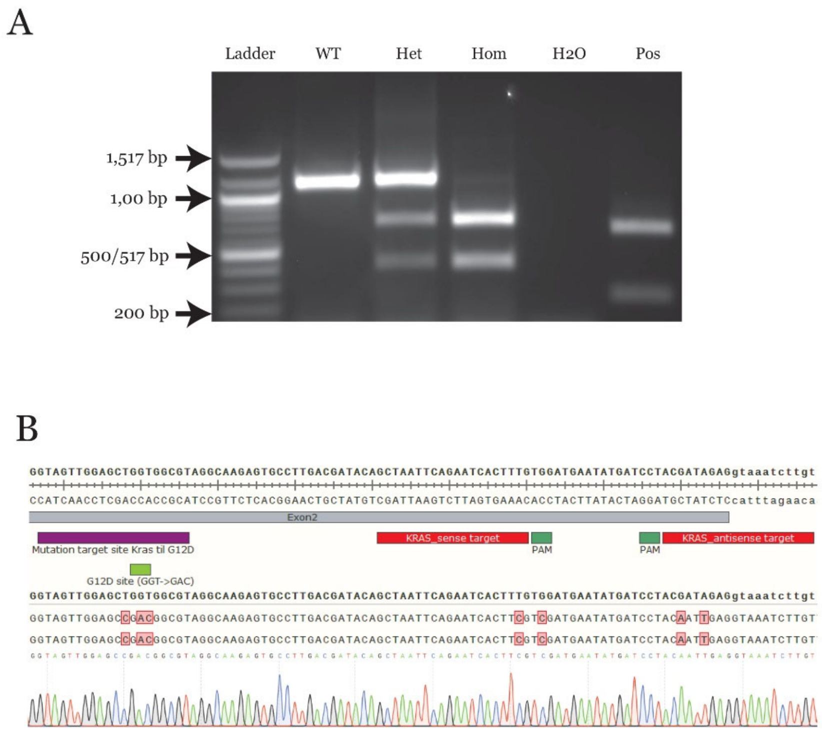

Figure S3. Introduction of KRAS^{G12D} point mutation by HDR. (**A**) The introduction of the KRAS^{G12D} point mutation by CRISPR HDR was assessed in single clones. Fibroblast from Cas9 transgene pic was co-transfected with sgRNA for KRAS and a KRAS^{G12D} repair template. Introduction of KRAS^{G12D} generated a restriction site and clones were analysed by PCR followed by a enzymatic digestion for the present of the new restriction site; KRAS^{G12D} mutated clones revealed two bands and non-mutated clones had a single band. Heterozygotes revealed three bands, one corresponding to the WT band and two to the cleaved mutated allele. Positive control was the KRASG12D template. (**B**) Sanger sequencing verified that the DNA sequence of the KRAS gene was identical to the sequence of the KRAS^{G12D} HDR template. Western Blot images can be found in Figure S7.

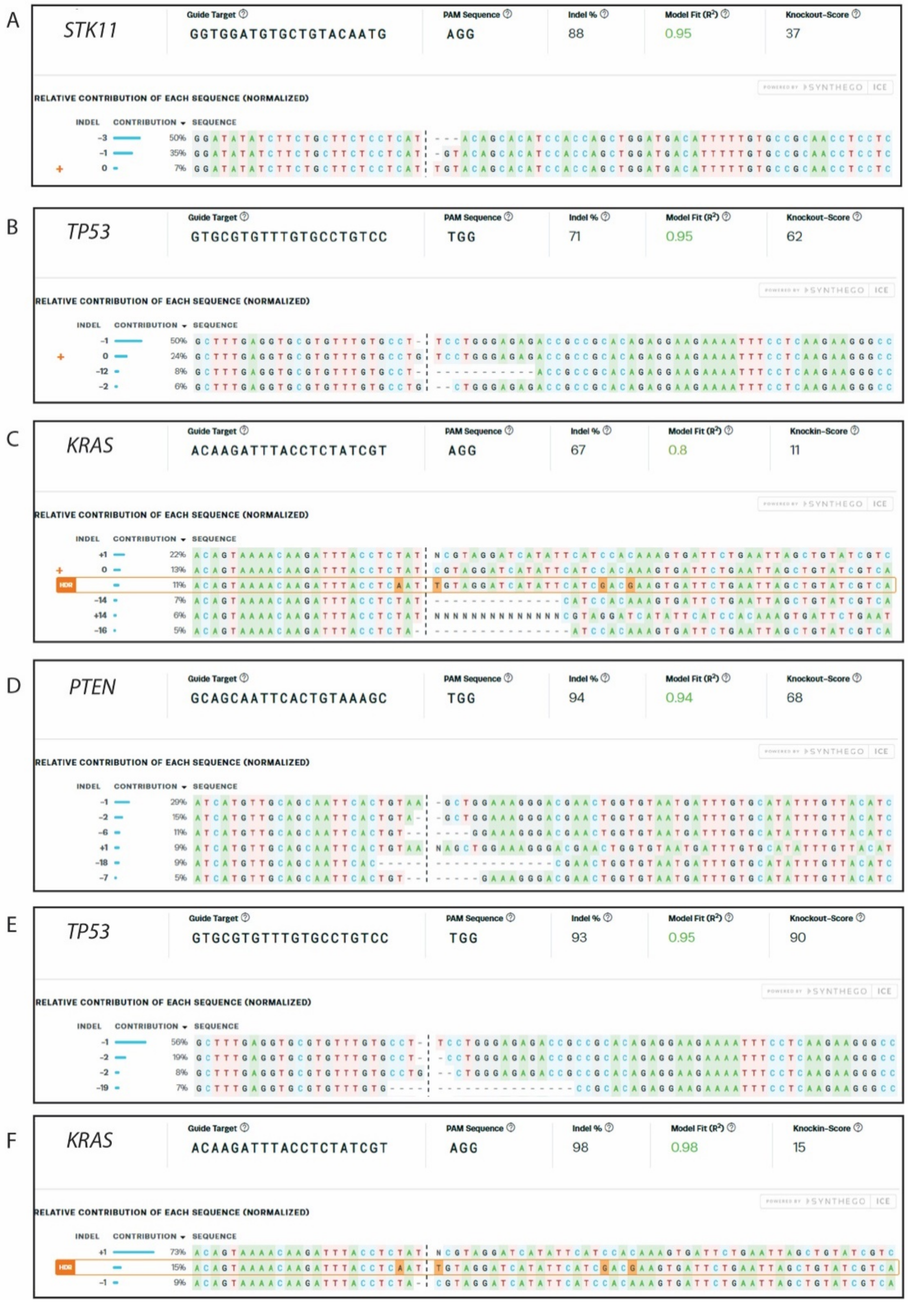

Figure S4. ICE analysis for AAV induces mutations in vitro. (**A**) STK11 mutations in SKT_AAV2H22 transduced Cas9 fibroblasts. (**B**) TP53 mutations in SKT_AAV2H22 transduced Cas9 fibroblasts. (**C**) KRAS mutations and KRAS^{G12D} repair in SKT_AAV2H22 transduced Cas9 fibroblasts. (**D**) PTEN mutations in PTK_AAV9 transduced Cas9 fibroblasts. (**E**) TP53 mutations in PTK_AAV9 transduced Cas9 fibroblasts. (**F**) KRAS mutations and KRAS^{G12D} repair in PTK_AAV9 transduced Cas9 fibroblasts.

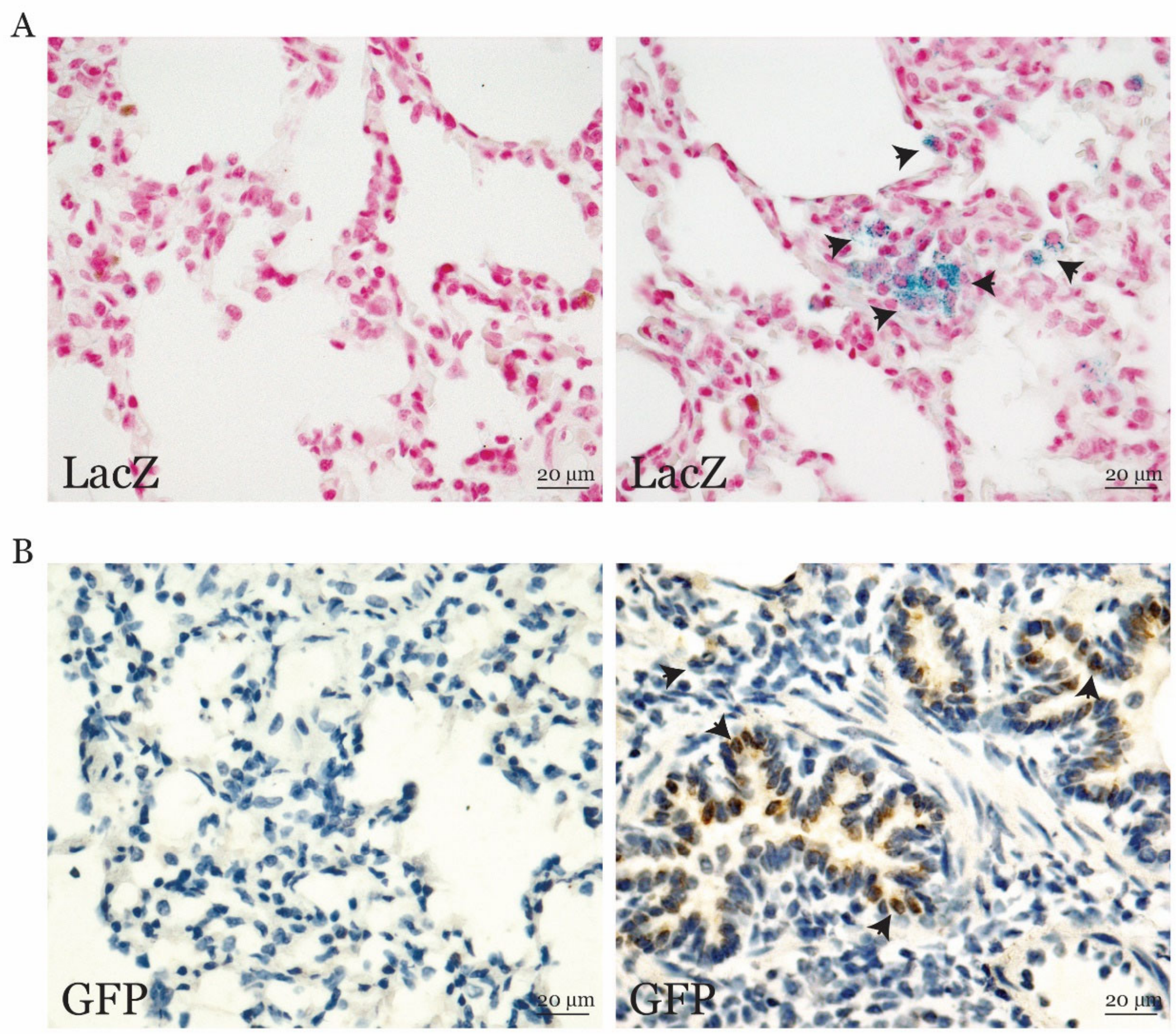

Figure S5. AAV targeting of pig lung epithelium. (**A**) β-galactosidase for LacZ in LacZ_AAV2H22 treated (right) and non-treated (left) porcine lungs. (**B**) Immunohistochemically staining for GFP in GFP_AAV9 treated (right) and non-treated (left) porcine lungs. Arrowheads mark positive cells.

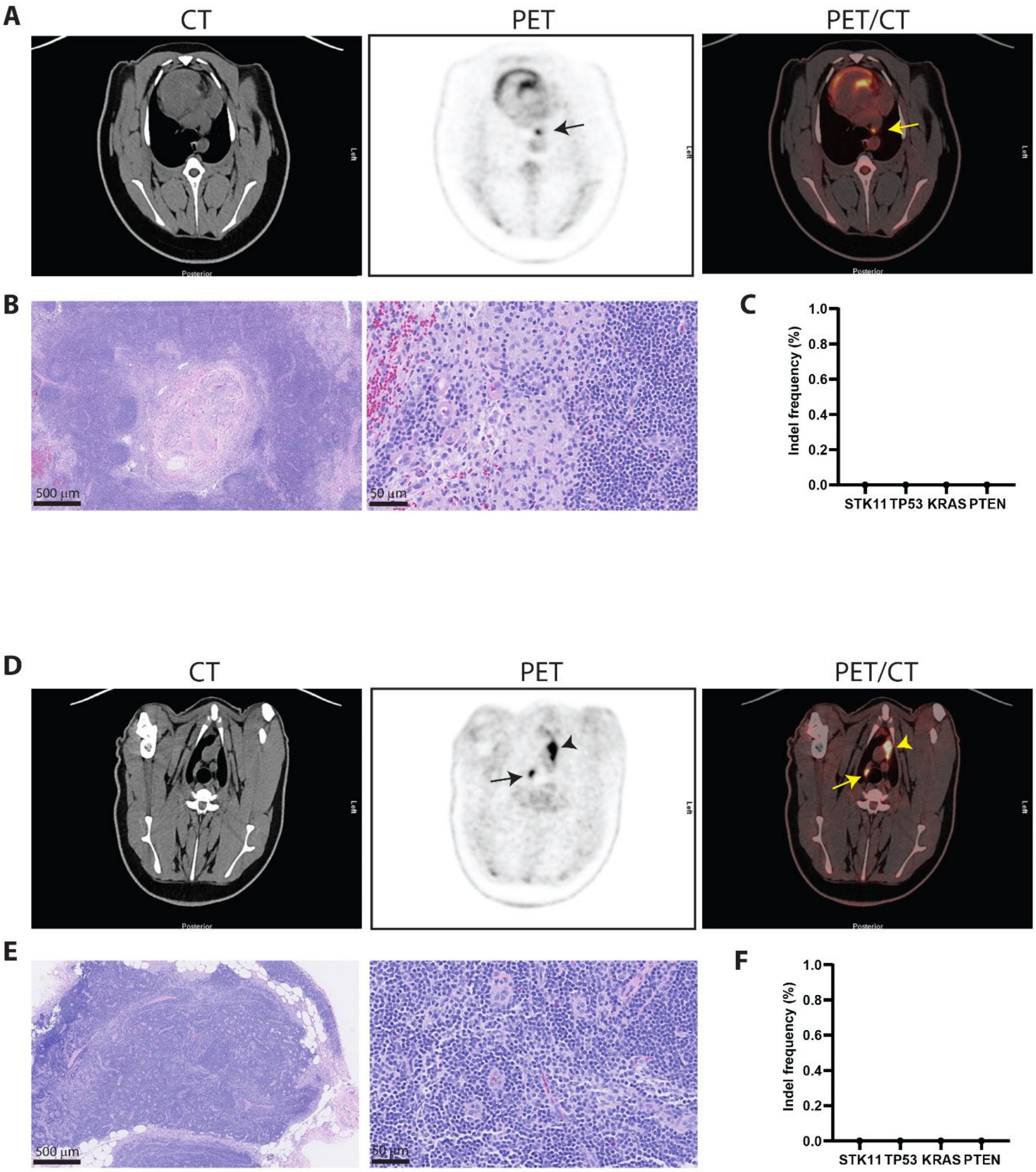

Figure S6. Activated lymph nodes. Activated lymph nodes were detected by increased ^{18}F-FDG signal by PET/CT scans. (**A**,**D**) PET/CT scans from two Cas9 mini pigs 18 months post AAV transduction. Arrows mark the activated lymph nodes and arrowhead mark thymus. Representative images are shown. (**B**,**E**) Histological sections from activated lymph nodes stained with H&E. (**C**,**F**) Indel analysis for CRISPR induced mutations in the four target genes. No Indels were detected by the Sanger sequencing.

SB - Figure 1

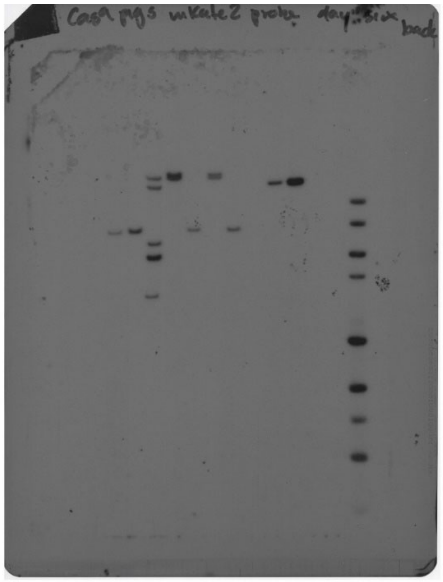

Viral DNA - Figure 4

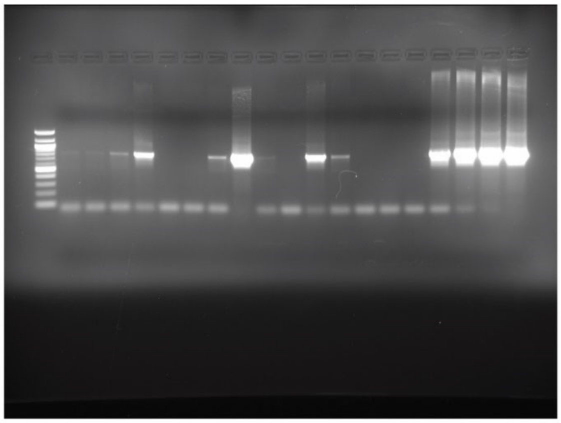

Recombination
Figure 4

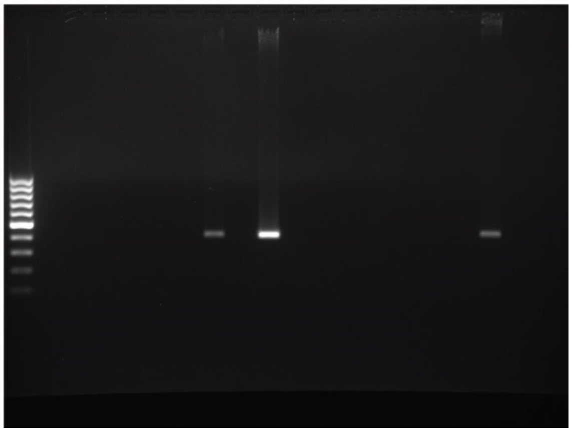

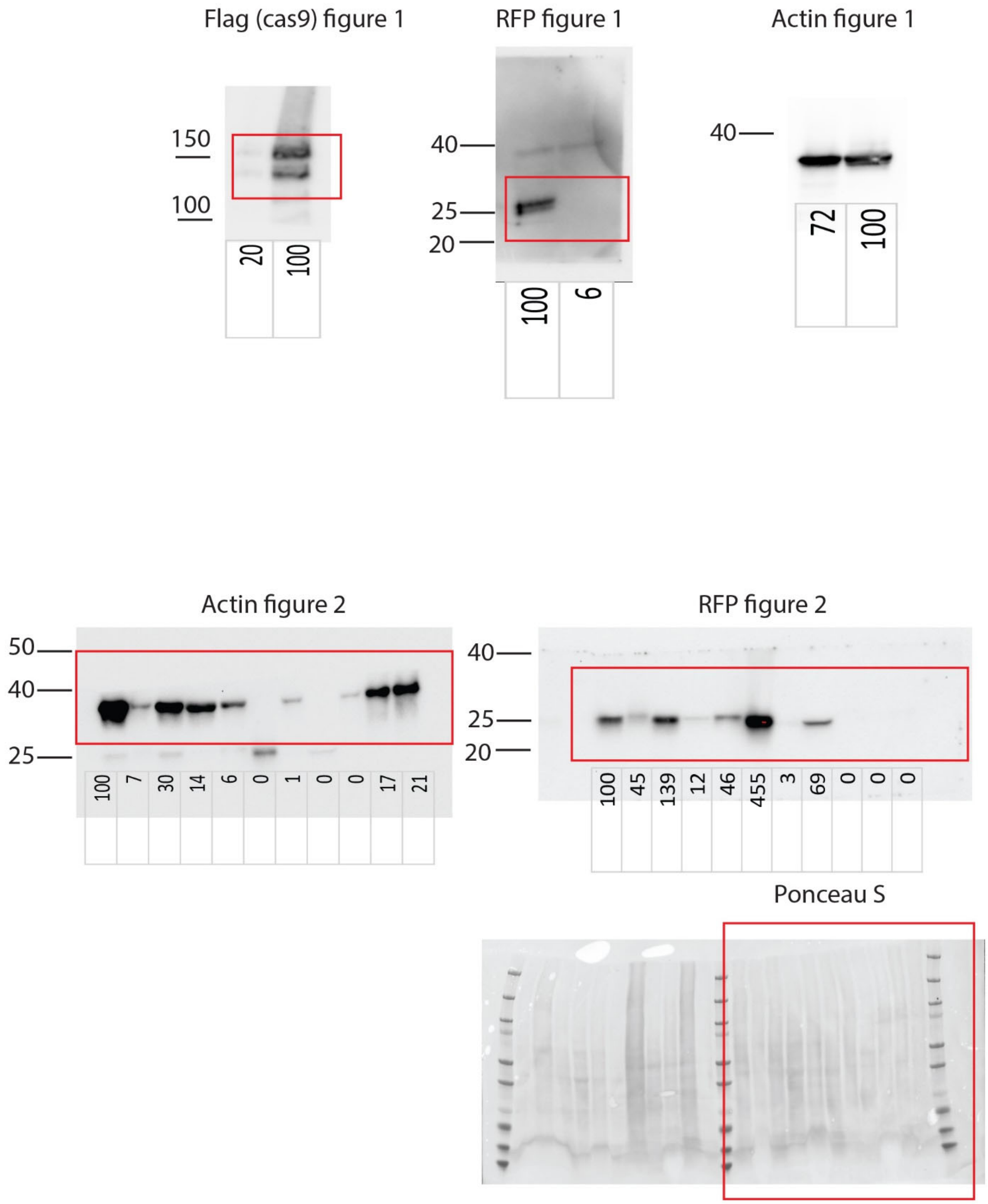
Flag (cas9) figure 1
150
100
20
100
RFP figure 1
40
25
20
100
6
Actin figure 1
40
72
100
Actin figure 2
50
40
25
100 7 30 14 6 0 1 0 0 17 21
RFP figure 2
40
25
20
100 45 139 12 46 455 3 69 0 0 0
Ponceau S

Figure 3

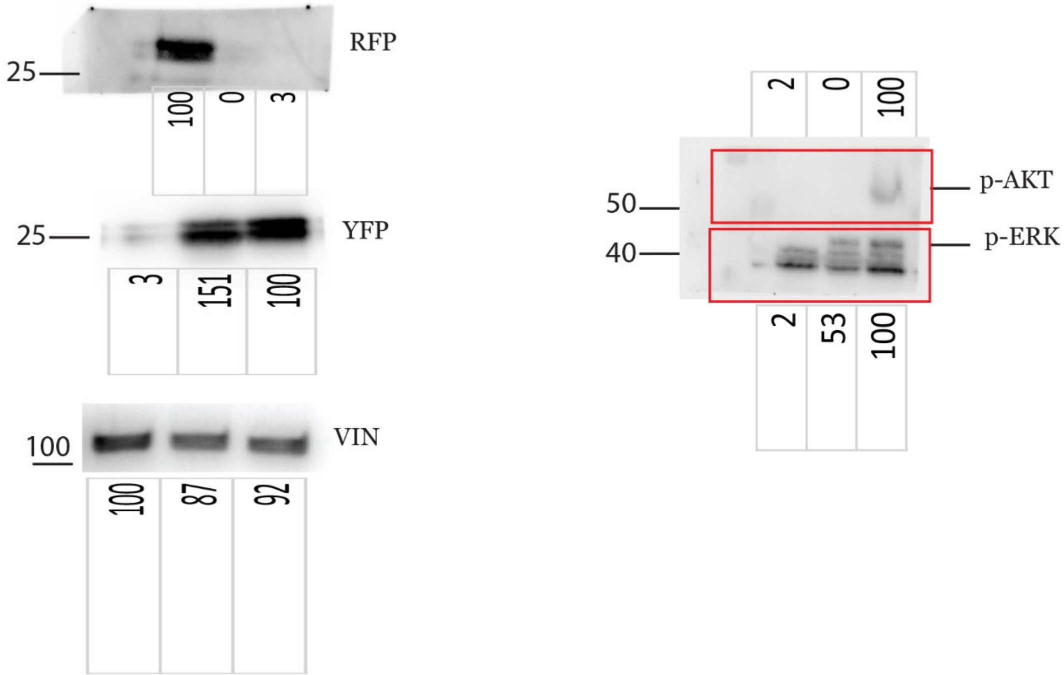

Flag (cas9) figure 5

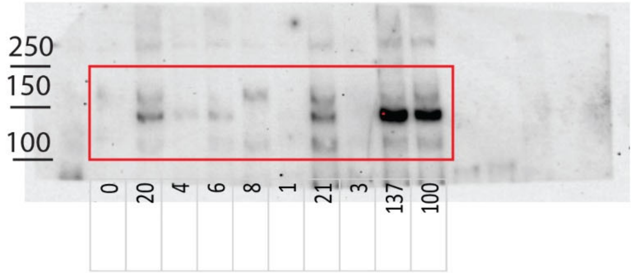

Actin figure 5

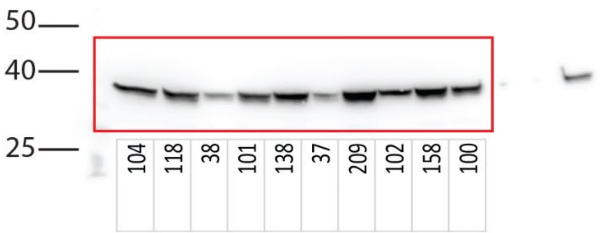

Figure S7. Uncropped Western Blot and Southern Blot images.

Table S1. Primers.

Primer:	Sequence:
KRAS_sgRNA_F	caccGACAAGATTTACCTCTATCGT
KRAS_sgRNA_R	aaacACGATAGAGGTAAATCTTGTC
KRAS_seq_F	AGTGAAGTCATGGCCCACTC
KRASG12D_seq_F	AGGCCTGCTGAAAATGACTGA
KRAS_seq_R	AAACACCAAAACCCCATACG
TP53_sgRNA_F	caccGTGCGTGTTTGTGCCTGTCC
TP53_sgRNA_R	aaacGGACAGGCACAAACACGCAC
TP53_seq_F	GGCTTCTTGATCAGCTGGAG
TP53_seq_R	TCGCCATCCAGTGGCTTCTTC
STK11_sgRNA_F	caccGGTGGATGTGCTGTACAATG
STK11_sgRNA_R	aaacCATTGTACAGCACATCCACC
STK11_seq_F	ATTCTTTGGGGCTGCTCTCC
STK11_seq_R	AGCCATAGAGGGGGCAACTA
PTEN_sgRNA_F	caccGCAGCAATTCACTGTAAAGC
PTEN_sgRNA_R	aaacGCTTTACAGTGAATTGCTGC
PTEN_seq_F	TTGGCCTCCCTATCTAATGG
PTEN_seq_R	CTCTGGTCCTTACTTCCCCAT
NOTCH1_sgRNA_F	caccGCGTAGTCCACCACACCGCCG
NOTCH1_sgRNA_R	aaacCGGCGGTGTGGTGGACTACGC
NOTCH1_seq_F	GGGTACAGTTCCGTGGTGTT
NOTCH1_seq_R	GGCCCTTGGGTGTCCTTAC
Activation_primary_F	CAGCCATTGCCTTTTATGGT
Activation_primary_R	TGTCGCCCTCGAACTTCAC
Activation_nested_F	GCTGGTTGTTGTGCTGTCTC
Activation_nested_R	AAGTCGTGCTGCTTCATGTG
AAV_presence_F	ATCAGCAAGGAGATGATCGC
AAV_presence_R	TTAGCTGTATCGTCAAGGCACTC
AAV_titration_F	GGAACCCCTAGTGATGGAGTT
AAV_titration_R	CGGCCTCAGTGAGCGA
